# CORRIGENDUM

**DOI:** 10.1002/1348-9585.12301

**Published:** 2021-12-09

In Al‐Qadi MM,^1^ the following error was published in Figure 1.

**In the bottom slab, “Included,” box “Studies included in the qualitative synthesis (*n* = 34).”**

The text was incorrect and should have read:

Studies included in the analysis (*n* = 34).

The corrected Figure 1 is below:

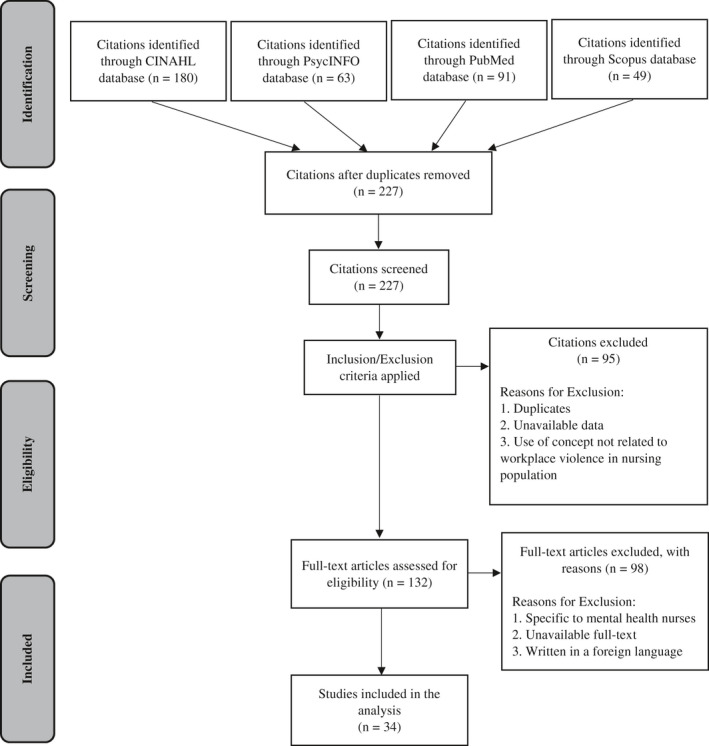

The author apologizes for this error.
